# Association between Markers of Fatty Liver Disease and Impaired Glucose Regulation in Men and Women from the General Population: The KORA-F4-Study

**DOI:** 10.1371/journal.pone.0022932

**Published:** 2011-08-05

**Authors:** Ina-Maria Rückert, Margit Heier, Wolfgang Rathmann, Sebastian E. Baumeister, Angela Döring, Christa Meisinger

**Affiliations:** 1 Institute of Epidemiology II, Helmholtz Zentrum München - German Research Center for Environmental Health (GmbH), Neuherberg, Germany; 2 MONICA/KORA Myocardial Infarction Registry, Central Hospital of Augsburg, Augsburg, Germany; 3 Institute of Biometrics and Epidemiology, German Diabetes Center, Leibniz Center for Diabetes Research at Heinrich Heine University Düsseldorf, Düsseldorf, Germany; 4 Institute for Community Medicine, University of Greifswald, Greifswald, Germany; 5 Institute of Epidemiology I, Helmholtz Zentrum München - German Research Center for Environmental Health (GmbH), Neuherberg, Germany; University Institute of Social and Preventive Medicine, Switzerland

## Abstract

**Objective:**

To investigate whether the elevated liver enzymes gamma-glutamyltransferase (GGT), glutamate-pyruvate transaminase (GPT), glutamate-oxalacetate transaminase (GOT) and alkaline phosphatase (AP) and non-alcoholic fatty liver disease (NAFLD) respectively are independently associated with pre-diabetic states, namely impaired fasting glucose (IFG) and impaired glucose tolerance (IGT) or known and newly diagnosed diabetes (NDD), in men and women from the general German population.

**Methods:**

The study was based on 3009 subjects (1556 females, 1453 males) aged 32 to 81 years who participated in the KORA-F4-Study in 2006/2008 in Augsburg, Southern Germany. All non-diabetic participants underwent an oral glucose tolerance test to assess disturbances in glucose metabolism. NAFLD was estimated by liver enzyme concentrations and the Bedogni Fatty Liver Index (FLI).

**Results:**

229 participants (7.6%) reported known diabetes, 106 had NDD (3.5%), 107 (3.6%) had IFG, 309 (10.3%) had IGT, 69 (2.3%) were affected with both metabolic disorders (IFG/IGT) and 74 (2.5%) could not be classified. GGT and GPT were significantly elevated in persons with pre-diabetes and diabetes (GGT in diabetic persons OR = 1.76, [1.47–2.09], in IFG OR = 1.79 [1.50–2.13], GPT in diabetic persons OR = 1.51, [1.30–1.74], in NDD OR = 1.77 [1.52–2.06]), GOT and AP only inconsistently in some pre-diabetes groups. The effects were sharpened in models using an increase of two or three out of three enzymes as an estimate of fatty liver and especially in models using the FLI. Overall frequency of NAFLD applying the index was 39.8% (women: 27.3% and men: 53.2%). In participants with fatty liver disease, the OR for NDD adjusted for sex and age was 8.48 [5.13–14.00], 6.70 [3.74–12.01] for combined IFG and IGT and 4.78 [3.47–6.59] for known diabetes respectively.

**Conclusions:**

Elevated GGT and GPT–values as well as estimates of fatty liver disease are significantly associated with pre-diabetes and diabetes and thus very useful first indicators of a disturbed glucose metabolism.

## Introduction

First described by Ludwig in 1980 [1], non-alcoholic fatty liver disease (NAFLD) is believed to be one of the most common causes of chronic liver disease in the Western world today. The prevalence is likely to parallel the increasing prevalence of diabetes, obesity, and other components of the metabolic syndrome and varies remarkably depending on the population studied and the diagnostic criteria used. Recent assessments, summarized by Bellentani and Marino, amount from about 3–30% in population based samples to 70–90% in obese patients eg. from bariatric surgeries. About 30% of all adult Americans, 25% of adult Italians and 14% and 15% Chinese and Japanese adults respectively are affected [2].

The clinicopathologic syndrome of NAFLD encompassing a spectrum of conditions ranging from benign accumulation of fat in the liver to inflammatory steatohepatitis (NASH), fibrosis and cirrhosis, has been characterized in detail [3]–[6].

Although the simultaneous occurrence of type 2 diabetes with NAFLD or elevated liver enzymes is a frequent observation [7]–[10] and the role of insulin resistance has been reviewed [11], few studies with selected (clinical) populations have so far described the association between liver enzymes as surrogate measurements of fatty liver disease and “pre-diabetes” [12]–[16].

Thus, the specific objective of the present article was to investigate the association between liver enzymes, fatty liver and type 2 diabetes mellitus (known and newly detected) as well as impaired glucose tolerance (IFG, impaired fasting glucose, IGT, impaired glucose tolerance and the combination of IFG and IGT) in the KORA (Cooperative Health Research in the Region of Augsburg) F4 study, including men and women from the general German population. The KORA study consists of an extensively phenotyped, population based sample of men and women with a wide age range, oral glucose tolerance tests (OGTT) and multiple clinical measurements and is therefore optimally applicative for confounder controlled analyses of associations concerning diabetes and other chronic diseases.

We applied the Fatty Liver Index (FLI) by Bedogni et al. (2006) [17] which was developed using ultrasound, is easy to adopt in clinical practice, and has independently been tested for its predictive ability concerning incident diabetes in a French study [18] and its association with insulin resistance in the Italian RISC Study [19].

## Methods

### Ethics statement

The investigations were carried out in accordance with the Declaration of Helsinki, including written informed consent of all participants. All study methods were approved by the Ethics committee of the “Bayerische Landesärztekammer” Munich.

### The KORA F4 study

The KORA F4 study is a follow-up of the KORA S4 study, a population-based health survey conducted in the city of Augsburg and two surrounding counties between 1999 and 2001. A total sample of 6640 subjects was drawn from the target population consisting of all German residents of the region aged 25 to 74 years.

Of all 4261 participants of the S4 baseline study, 3080 also participated in the 7-year follow-up F4 study. Persons were considered ineligible for F4 if they had died in the meantime (N = 176, 4%), lived outside the study region or were completely lost to follow-up (N = 206, 5%), or had demanded deletion of their address data (N = 12, 0.2%). Of the remaining 3867 eligible persons, 174 could not be contacted, 218 were unable to come because they were too ill or had no time, and 395 were not willing to participate in this follow-up, giving a response rate of 79.6%. After exclusion of participants with missing data in any of the variables used in our analyses (N = 71), 3009 subjects (1556 females (51.7%) and 1453 males) remained. Thus, all models are based on complete case analyses.

### Data collection

Information on socio-demographic variables, smoking habits, physical activity, medication use, alcohol consumption and household characteristics were gathered by trained medical staff during a standardized interview. Educational attainment was estimated by the number of school years completed by the participant. The assessment of alcohol intake (grams per day) was based on weekday and weekend consumption of beer, wine and spirits and study participants provided information on their smoking behaviour (never, past, current). Furthermore, they underwent an extensive standardized medical examination including the collection of blood samples. All measurement procedures were described in detail elsewhere [20]. Waist and hip circumferences were measured with a measurement tape in front of a whole body mirror: waist midway between the lowest rib and the iliac crest and hip at the greatest girth at the level of the buttocks, between the iliac crest and the crotch. Body mass index (BMI) was calculated as weight in kilograms divided by height in square metres. Actual hypertension was defined as blood pressure values ≥140/90 mmHg and/or use of antihypertensive medication given that the subjects were aware of being hypertensive. Individuals who participated in leisure time physical training during summer or winter for at least one hour per week were classified as being physically active.

Men who consumed more than 40 g (N = 412) and women who consumed more than 20 g alcohol per day (N = 290) or reported past abuse of alcohol within the last 5 years were regarded as at-risk alcohol drinkers and excluded from some of the models in order to rule out alcoholic fatty liver disease.

Fatty liver disease was defined using three different approaches:

Definition A: Increase of at least two out of the three enzymes GGT (gamma-glutamyltransferase), GPT (glutamate-pyruvate transaminase) and GOT (glutamate-oxalacetate transaminase) above the upper gender-specific medical reference values, which were taken from Roche/Hitachi cobas® enzyme laboratory manuals (2007) which included consensus values according to Thomas et al. (2005) [21] (GGT in men 1.0 µkat/l, in women 0.67 µkat/l, GPT and GOT in men 0.83 µkat/l, in women 0.58 µkat/l).

Definition B: Increase of at least two out of the three enzymes GGT, GPT and GOT in the upper population quartile of the enzyme distribution (GGT in men 0.89 µkat/l, in women 0.50 µkat/l, GPT in men 0.60 µkat/l, in women 0.39 µkat/l and GOT in men 0.53 µkat/l, in women 0.44 µkat/l). This established cutpoint was chosen in order to be able to compare our results with similar studies.

Definition C: Calculation of the Fatty Liver-Index (FLI) according to Bedogni, 2006 [17]:
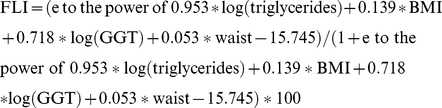
The FLI varies between 0 and 100. The score 0–29 rules out fatty liver disease and 30–59 is defined as unclear. We combined these two groups. A score of greater or equal 60 points was considered as ruling in fatty liver disease. In the original study, this cutpoint had a sensitivity of 61% and a specificity of 86% to correctly discern patients with and without NAFLD.

### OGTT-measurement to detect impaired glucose metabolism

Previously diagnosed diabetes was defined as validated physician diagnosis or current intake of anti-diabetic agents. After an overnight fast of at least 8 hours, all non-diabetic participants underwent a standard 75-g oral glucose tolerance test (OGTT) [20]. Blood was collected without stasis, refrigerated to 4–8°C and shipped on refrigerant packaging within 4 to 6 hours to the laboratory. Newly diagnosed diabetes (NDD), IFG, IGT, and normal glucose tolerance (NGT) were defined according to the 1999 WHO diagnostic criteria based on both fasting and post-challenge glucose values [22]. Thus, a participant had NGT if the fasting glucose value was <110 mg/dl and the 2 h-value <140 mg/dl. NDD was diagnosed, if fasting glucose was >125 mg/dl and post challenge glucose ≥200 mg/dl. IGT was defined as having a fasting level of <126 mg/dl and a 2 h post glucose load of between 140 and 200 mg/dl and IFG as having a fasting level of between 110 and 125 mg/dl and a 2 h measurement of <140 mg/dl.

We used the original IFG criteria (110 to 125 mg/dl or 6.1 to 6.9 mmol/l respectively) for the present analysis, as recommended by the European Diabetes Epidemiology Group [23]. The pre-diabetic state was defined as having either IGT, IFG or both IGT and IFG. Participants were classified with unknown glucose status, if they had given inconsistent information about being diabetic or if their OGT test could not be conducted because of contraindications like pregnancy or no previous fasting period or could not be completed correctly (because of nausea, vomiting etc.).

### Clinical chemical measurements

A fasting venous blood sample was obtained from all study participants while sitting. Blood glucose was analysed using a hexokinase method (Gluco-quant, Roche Diagnostics, Mannheim, Germany). Glycated hemoglobin A1c (HbA1c)-values were assessed with a turbidimetric immunological assay (Tina-quant, Roche Diagnostics) and plasma C-reactive protein (CRP) concentrations were measured using a latex enhanced nephelometric assay run on a BN II analyser (Dade Behring, Marburg, Germany).

The liver enzymes GGT, GOT, GPT and AP (alkaline phosphatase) were analyzed according to the recommendations of the International Federation of Clinical Chemistry (IFCC) from 1983 (confirmed and extended in 2002) [21], [24]–[29], including optimization of substrate concentrations, employment of NaOH, glycylglydine buffer and sample start. Pyridoxal phosphate was applied in the assessment of GOT and GPT. Total cholesterol was measured with the CHOD-PAP method using a CHOL Flex device (Dade Behring), high-density lipoprotein (HDL-) cholesterol was also determined using the CHOD-PAP method but after selective release of HDL cholesterol on an AHDL Flex device (Dade Behring). Triglycerides were measured in an enzymatic colour test with the GPO-PAP method on a TGL Flex (Dade Behring) and serum uric acid was determined on an URCA Flex (Dade Behring) also applying an enzymatic colour test and the uricase method.

### Statistical Analyses

Continuous variables are given with medians and interquartile ranges because of skewed distributions. Between-group bivariable comparisons were performed with the Mann-Whitney-Wilcoxon test (continuous variables) and multinomial unadjusted logistic regression (categorical variables) using the NGT-group as the reference. Variance inflation analysis revealed no collinearity factors greater than 5, but the Spearman's rho coefficients for GGT vs. GPT, GOT vs. GPT and TC/HDL-cholesterol ratio vs. triglycerides were greater than 0.5.

Odds ratios (OR) are given per one SD increment in enzyme concentration, calculated from the subpopulation used in the respective model. Two multinomial models using the SAS PROC LOGISTIC procedure were created for each liver enzyme and the three fatty liver concepts respectively, one only adjusted for sex and age, the other for all relevant co-variables. The covariables used included age (years), sex (male/female), education (years), BMI (kg/m2), hypertension (≥140/90 or taking medication, see definition above), TC/HDL-cholesterol ratio (no unit), uric acid (µmol/l), CRP (mg/l), alcohol intake/past abuse (>20 g/day in females, >40 g/day in males, see definition above), smoking status (non-smoker/current or ex-smoker), and physical activity (active/inactive).

BMI was used as a categorical variable for BMI effect modification analyses. To categorize BMI we used the WHO international classification (1995) [30].

Moreover, sensitivity analyses were done by excluding all study participants with enzyme values above recent medical reference values (N = 711), excluding participants with alcohol intake >20 (females) or 40 g/day (males) or past abuse (N = 702), or participants using prescription medication (N = 2342) respectively. Interaction of liver enzymes/fatty liver and sex was tested by including interaction terms (enzyme*sex or fatty liver*sex) together with main factors in multinomial logistic models. Goodness-of-fit was estimated using Pearson tests, Akaike's information criterion (AIC) and Pseudo-R-Square.

A value of p<0.05 was considered statistically significant. All analyses were performed with SAS software version 9.1 (SAS Institute Inc., Cary, NC, USA.).

## Results

### Study characteristics

In total 229 participants (7.6%, 9.3% of men and 6.0% of women) reported known diabetes, 106 had NDD (4.2% of men, 2.9% of women), 107 (3.6%) had IFG, 309 (10.3%) had IGT, 69 (2.3%) were affected with both metabolic disorders (IFG/IGT) and 74 (2.5%) could not be classified. This last class mostly resembled the NGT group (2115, 70.3%) in our models – thus both could probably have been considered as one, but we chose to look at them separately.

Table 1 shows study characteristics stratified for glucose subgroup. Most important, GGT and GPT were significantly increased in all pre-diabetes and the diabetes subgroup compared to persons with NGT. GOT was not significantly increased in persons with known diabetes and unknown status, but all other groups. A significant elevation of AP values was observed in persons with IFG, IGT, NDD and known diabetes, but not in the group of participants who had IFG/IGT and the group with unknown glucose tolerance status.

**Table 1:** Study characteristics by NGT, IFG-NGT, NFG-IGT, IFG/IGT, NDD, known Diabetes and unknown glucose tolerance status.

N = 3009	NGT	IFG-NGT	NFG-IGT	IFG/IGT	NDD	Known Diabetes	Unknown
	(n = 2115)	(n = 107)	(n = 309)	(n = 69)	(n = 106)	(n = 229)	(n = 74)
**GGT**	0.39	0.60	0.51	0.56	0.70	0.57	0.41
**(µkat/l)**	(0.35)	(0.74)	(0.49)	(0.50)	(0.52)	(0.53)	(0.54)
		<.0001	<.0001	<.0001	<.0001	<.0001	0.1372
**GOT**	0.40	0.46	0.44	0.44	0.46	0.42	0.40
**(µkat/l)**	(0.14)	(0.16)	(0.17)	(0.14)	(0.22)	(0.15)	(0.13)
		<.0001	<.0001	0.0002	<.0001	0.0873	0.7903
**GPT**	0.34	0.45	0.40	0.44	0.47	0.38	0.33
**(µkat/l)**	(0.22)	(0.27)	(0.26)	(0.21)	(0.37)	(0.26)	(0.24)
		<.0001	<.0001	<.0001	<.0001	0.0006	0.6530
**AP**	1.07	1.21	1.17	1.17	1.23	1.15	1.11
**(µkat/l)**	(0.42)	(0.51)	(0.39)	(0.40)	(0.31)	(0.44)	(0.38)
		<.0001	<.0001	0.0864	<.0001	0.0005	0.6459
**FLI**	34.2	77.9	67.0	82.2	83.1	80.0	43.7
	(51.6)	(35.4)	(43.1)	(31.8)	(25.5)	(38.7)	(56.9)
		<.0001	<.0001	<.0001	<.0001	<.0001	0.0229
**Age**	52.0	62.0	65.0	65.0	65.0	69.0	53.5
**(years)**	(21.0)	(17.0)	(17.0)	(10.0)	(18.0)	(11.0)	(28.0)
		<.0001	<.0001	<.0001	<.0001	<.0001	0.0818
**Female**	54.5	33.6	53.7	39.1	43.0	41.1	47.3
**(%)**		<.0001	0.7936	0.0129	0.0159	0.0001	0.2220
**Education**	40.4	49.5	55.3	43.5	54.7	64.6	48.7
**(< = 10 years, %)**		0.0614	<.0001	0.6059	<.0001	<.0001	0.1563
**Living alone**	24.2	23.4	24.9	29.0	27.4	25.8	27.0
**(%)**		0.8424	0.7855	0.3639	0.4613	0.6024	0.5787
**BMI**	26.0	29.1	29.3	30.9	30.4	30.6	26.3
**(kg/m2)**	(5.3)	(5.2)	(5.8)	(6.4)	(5.1)	(7.1)	(5.9)
		<.0001	<.0001	<.0001	<.0001	<.0001	0.3230
**Waist** **cirumference**	90.1	102.2	98.7	104.1	103.5	104.2	92.3
**(cm)**	(17.5)	(14.0)	(15.4)	(19.7)	(15.6)	(16.4)	(16.7)
		<.0001	<.0001	<.0001	<.0001	<.0001	0.0156
**Hip** **circumference**	104.0	109.1	108.3	108.7	109.5	109.7	103.4
**(cm)**	(9.9)	(11.2)	(11.8)	(11.4)	(9.7)	(13.4)	(10.9)
		<.0001	<.0001	<.0001	<.0001	<.0001	0.5824
**Waist-Hip Ratio**	0.86	0.94	0.91	0.95	0.94	0.96	0.88
	(0.12)	(0.10)	(0.12)	(0.08)	(0.10)	(0.10)	(0.14)
		<.0001	<.0001	<.0001	<.0001	<.0001	0.0083
**Systolic blood pressure**	117.5	128.0	125.0	134.5	132.0	131.0	121.3
**(mm HG)**	(23.0)	(20.5)	(24.5)	(19.0)	(23.5)	(24.0)	(24.0)
		<.0001	<.0001	<.0001	<.0001	<.0001	0.0414
**Diastolic blood pressure**	74.0	77.0	74.5	81.0	76.0	74.0	75.3
**(mm HG)**	(13.0)	(14.5)	(13.0)	(10.5)	(13.0)	(13.5)	(13.5)
		0.0094	0.1232	<.0001	0.0162	0.6681	0.8405
**Current hypertension**	27.5	53.3	54.7	69.6	77.4	79.5	39.2
**(%)**		<.0001	<.0001	<.0001	<.0001	<.0001	0.0288
**Total cholesterol**	213.0	221.0	225.0	222.0	214.0	198.0	220.5
**(mg/dL)**	(50.0)	(47.0)	(56.0)	(50.0)	(44.0)	(53.0)	(52.0)
		0.0120	<.0001	0.0117	0.3936	<.0001	0.2387
**HDL cholesterol**	56.0	49.0	53.0	47.0	45.5	48.0	53.0
**(mg/dL)**	(20.0)	(18.0)	(19.0)	(17.0)	(17.0)	(15.0)	(25.0)
		<.0001	0.0013	<.0001	<.0001	<.0001	0.0992
**TC/HDL ratio**	3.8	4.5	4.2	4.7	4.6	4.2	4.1
	(1.5)	(1.6)	(1.5)	(1.8)	(1.6)	(1.4)	(1.4)
		<.0001	<.0001	<.0001	<.0001	<.0001	0.0327
**TC/HDL ratio**≥**5 (%)**	16.5	31.8	24.9	43.5	36.8	22.7	23.0
		<.0001	0.0003	<.0001	<.0001	0.0185	0.1451
**Triglycerides**	94.0	129.0	119.0	171.0	147.0	137.0	111.0
**(mg/dL)**	(69.0)	(86.0)	(78.0)	(120.0)	(124.0)	(94.0)	(78.0)
		<.0001	<.0001	<.0001	<.0001	<.0001	0.0094
**HbA1c**	5.4	5.7	5.6	5.8	6.1	6.7	5.4
**(%)**	(0.5)	(0.4)	(0.5)	(0.5)	(0.8)	(1.2)	(0.5)
		<.0001	<.0001	<.0001	<.0001	<.0001	0.4694
**Uric acid**	4.9	6.1	5.5	6.0	5.8	6.0	5.2
**(µmol/l)**	(1.8)	(2.2)	(1.9)	(1.6)	(1.7)	(2.1)	(2.2)
		<.0001	<.0001	<.0001	<.0001	<.0001	0.2394
**CRP**	1.0	1.5	1.7	2.4	2.9	1.9	1.2
**(mg/l)**	(1.6)	(2.9)	(3.1)	(2.8)	(4.4)	(2.8)	(2.5)
		<.0001	<.0001	<.0001	<.0001	<.0001	0.0573
**Alcohol intake**	5.7	9.5	5.7	13.0	8.6	2.9	5.1
**(g/day)**	(20.0)	(30.3)	(20.0)	(24.6)	(25.7)	(17.6)	(22.9)
		0.1250	0.5364	0.1171	0.3412	0.0029	0.6436
**Alcohol >** **that 20 g(f) or 40 g(m)/day or past abuse**	23.4	32.7	20.4	29.0	25.5	20.1	21.6
**(%)**		0.3790	0.1787	0.2973	0.8885	0.0049	0.5775
**Current or ex-smoking**	56.2	64.5	47.9	50.7	58.5	58.1	54.1
**(%)**		0.0938	0.0062	0.3665	0.6453	0.5896	0.7125
**Physically active during leisure time**	58.2	45.8	49.5	53.6	48.1	36.2	52.7
**(%)**		0.0124	0.0043	0.4534	0.0423	<.0001	0.3511

Data is expressed as Median (IQ-range 25–75) and p-value or % and p-value respectively.

Multinomial unadjusted logistic regression analyses and Mann-Whitney-Wilcoxon were used for bivariable comparisons with NGT as reference.

NGT: normal glucose tolerance, IFG: impaired fasting glucose, NFG: normal fasting glucose, IGT: impaired glucose tolerance, NDD: newly diagnosed diabetes, GGT: gamma-glutamyltransferase, GOT: glutamate-oxalacetate transaminase, GPT: glutamate-pyruvate transaminase, AP: alkaline phosphatase, FLI: Fatty Liver Index, BMI: Body Mass Index, HDL: high density lipoprotein, TC: total cholesterol, HbA1c: glycated hemoglobin, CRP: C-reactive protein.

Other study characteristics also varied according to glucose subgroup: participants affected with glucose disturbances were significantly older than the healthy reference, males were more often affected, a significantly greater number of participants with less than 10 years education had IGT, NDD or known diabetes. BMI, waist, hip circumference and hypertension were markedly increased in all pre-/diabetes subgroups. Total cholesterol was higher in pre-diabetic than normoglycemic individuals and lower in patients with diabetes. HDL was decreased in all subgroups compared to the reference group. There was a clear increase in triglycerides, CRP, HbA1c and uric acid values in persons with glucose disturbances in comparison to persons with NGT.

Alcohol intake was lower in participants with known diabetes and otherwise not significantly different from the reference group. Frequency of current or ex-smoking was lower in IFG, but beyond that no association with diabetes or pre-diabetes could be detected. Finally, affected participants were significantly less physically active during their leisure time, especially persons with known diabetes.

### Single enzyme models

Results of age and sex as well as multivariable adjusted models with one liver enzyme as the main influencing factor are presented in table 2. GGT was associated with glucose metabolism to about the same extent (e.g. IFG: OR = 1.79, 95% CI: 1.50–2.13) in all subgroups. The effect remained significant after including multiple confounding variables in IFG, IGT, NDD, diabetes and the group with unknown status with a maintained tendency in the IFG/IGT group.

**Table 2:** Association of liver enzymes with glucose subgroups: Normal glucose tolerance NGT was used as reference, ORs are expressed per 1-SD increment in biomarker concentration.

N = 3009	IFG-NGT	NFG-IGT	IFG/IGT	NDD	Known Diabetes	Unknown
n (reference NGT) = 2115	(n = 107)	(n = 309)	(n = 69)	(n = 106)	(n = 229)	(n = 74)
**GGT**						
**Age-sex-** adjusted **OR**	**1.79**	**1.77**	**1.71**	**1.74**	**1.76**	**1.69**
[95% CI]	[1.50–2.13]	[1.50–2.10]	[1.34–2.18]	[1.42–2.13]	[1.47–2.09]	[1.35–2.13]
p-value	<.0001	<.0001	<.0001	<.0001	<.0001	<.0001
**Multivariable-** adjusted **OR**	**1.47**	**1.49**	**1.36**	**1.41**	**1.43**	**1.45**
[95% CI]	[1.23–1.77]	[1.26–1.76]	[0.99–1.86]	[1.11–1.79]	[1.19–1.73]	[1.19–1.77]
p-value	<.0001	<.0001	0.0606	0.0047	0.0002	0.0002
**GOT**						
**Age-sex-** adjusted **OR**	**1.39**	**1.34**	**1.25**	**1.40**	**1.18**	**1.03**
[95% CI]	[1.21–1.59]	[1.19–1.50]	[1.00–1.56]	[1.22–1.61]	[1.00–1.39]	[0.77–1.40]
p-value	<.0001	<.0001	0.0526	<.0001	0.0518	0.8297
**Multivariable-** adjusted **OR**	**1.25**	**1.22**	**1.07**	**1.27**	**1.01**	**1.01**
[95% CI]	[1.08–1.44]	[1.09–1.37]	[0.81–1.42]	[1.10–1.47]	[0.84–1.21]	[0.76–1.36]
p-value	0.0025	0.0005	0.6197	0.0014	0.9094	0.9254
**GPT**						
**Age-sex-** adjusted **OR**	**1.60**	**1.46**	**1.62**	**1.77**	**1.51**	**1.01**
[95% CI]	[1.37–1.87]	[1.29–1.66]	[1.34–1.97]	[1.52–2.06]	[1.30–1.74]	[0.75–1.36]
p-value	<.0001	<.0001	<.0001	<.0001	<.0001	0.9456
**Multivariable-** adjusted **OR**	**1.33**	**1.27**	**1.32**	**1.51**	**1.24**	**0.96**
[95% CI]	[1.12–1.58]	[1.12–1.45]	[1.05–1.67]	[1.27–1.79]	[1.06–1.45]	[0.71–1.30]
p-value	0.0009	0.0002	0.0186	<.0001	0.0077	0.7847
**AP**						
**Age-sex-**adjusted **OR**	**1.41**	**1.18**	**1.07**	**1.23**	**1.11**	**1.00**
[95% CI]	[1.19–1.66]	[1.05–1.34]	[0.83–1.38]	[1.01–1.48]	[0.95–1.28]	[0.78–1.28]
p-value	<.0001	0.0058	0.6122	0.0391	0.1900	0.9817
**Multivariable-** adjusted **OR**	**1.26**	**1.03**	**0.89**	**0.98**	**0.89**	**0.91**
(95% CI)	[1.04–1.52]	[0.90–1.17]	[0.67–1.19]	[0.79–1.23]	[0.75–1.05]	[0.70–1.19]
p-value	0.0161	0.6933	0.4252	0.8898	0.1556	0.5046

Multivariable-adjusted models included age, sex, education, BMI, hypertension, TC/HDL-cholesterol ratio, uric acid, CRP, alcohol intake, smoking status, and physical activity.

ORs were smaller, but also significant for GOT in IFG, IGT and NDD (OR for NDD: 1.40, 95% CI: 1.22–1.61). The other groups pointed in the same direction. Multivariable adjustment led to smaller though still significant effects. Similarly to GGT, in age and sex adjusted analyses GPT was significantly related to all glucose subgroups, except the group with unknown status (e.g. OR for IFG-IGT: 1.62, 95% CI: 1.34–1.97, OR for NDD: 1.77, 95% CI: 1.52–2.06). Only the group with IFG/IGT lost some significance after multivariable control (OR = 1.32, 95% CI: 1.05–1.67). Finally, AP showed least significant and smallest associations and only IFG remained significant in the extended model. The p-values for the interactions of GOT (p = 0.0159) and GPT (p = 0.0009) with sex were only statistically significant in the subgroup of known diabetes. GGT had some significant interaction with sex in the group with unknown status (p = 0.0401). If interaction models were adjusted for age, all enzyme-sex interactions became non-significant.

Data are not shown for further modified models stratified for sex and excluding participants with liver values above reference limits that were calculated to check sensitivity since the results were found to be quite stable.

All Pseudo-R-Square values were satisfactory between 0.2 and 0.4, Pearson tests and AICs confirmed appropriate goodness-of-fit of all models.

### Fatty liver estimates and models

Depending on the definitions described above, we got different estimates of fatty liver frequency in our study sample (Table 3). Applying the definition with reference values, 7.1% of the participants had fatty liver disease (NAFLD or AFLD) (6.9% women, 7.3% men). Using the quartile definition, the numbers increased to 22.6% overall and 22.4 in women, 22.8% in men respectively. Using the Bedogni Index even higher numbers resulted: there was a total of 39.8% fatty liver cases, 27.3% women and 53.2% men. The percentages slightly increased in women (28.2%) and decreased in men (51.3%) when subjects with significant alcohol consume were omitted from the calculations, indicating that the number of participants with alcoholic fatty liver disease was small.

**Table 3:** Association of fatty liver with glucose tolerance subgroups: Normal glucose tolerance was used as reference, fatty liver was defined in three different ways (A–C) as specified in the table.

N = 3009	IFG-NGT	NFG-IGT	IFG/IGT	NDD	Known Diabetes	Unknown
n(NGT) = 2115	(n = 107)	(n = 309)	(n = 69)	(n = 106)	(n = 229)	(n = 74)
A: Definition of fatty liver: two or three out of three enzymes (GGT, GOT and GPT) above upper reference value						
N (fatty liver, all participants) = 213 (7.1%)						
N (fatty liver, reference NGT) = 106 (5.0%)						
N (fatty liver)	14	34	6	16	30	7
(%)	(13.1)	(11.0)	(8.7)	(15.1)	(13.1)	(9.5)
**Age-sex-** adjusted **OR**	**3.38**	**2.83**	**2.29**	**4.42**	**4.08**	**2.05**
(95% CI)	[1.84–6.21]	[1.85–4.33]	[0.96–5.50]	[2.45–7.97]	[2.55–6.52]	[0.92–4.59]
p-value	<.0001	<.0001	0.0628	<.0001	<.0001	0.0806
**Multivariable-**adjusted **OR**	**2.10**	**2.11**	**1.34**	**2.63**	**2.64**	**1.91**
(95% CI)	[1.11–3.97]	[1.36–3.27]	[0.54–3.31]	[1.40–4.94]	[1.60–4.35]	[0.84–4.35]
p-value	0.0231	0.0009	0.5305	0.0027	0.0001	0.1234

B: Definition of fatty liver: two or three out of three enzymes above 75% quartiles of the population						
N (fatty liver, all participants) = 680 (22.6%)						
N (fatty liver, reference NGT) = 395 (18.7%)						
N (fatty liver)	37	102	23	45	59	19
(%)	(34.6)	(33.0)	(33.3)	(42.5)	(25.8)	(25.7)
**Age-sex-** adjusted **OR**	**2.50**	**2.29**	**2.49**	**3.77**	**1.87**	**1.50**
(95% CI)	[1.64–3.82]	[1.74–3.01]	[1.47–4.21]	[2.48–5.73]	[1.34–2.62]	[0.88–2.55]
p-value	<.0001	<.0001	0.0007	<.0001	0.0003	0.1386
**Multivariable-** adjusted **OR**	**1.67**	**1.79**	**1.53**	**2.50**	**1.30**	**1.41**
(95% CI)	[1.07–2.61]	[1.34–2.38]	[0.88–2.65]	[1.60–3.91]	[0.91–1.86]	[0.81–2.45]
p-value	0.0246	<.0001	0.1321	<.0001	0.1558	0.2227

C: Definition of fatty liver: according to Fatty Liver Index (Bedogni, 2006)						
N (fatty liver, all participants) = 1198 (39.8%)						
N (fatty liver, reference NGT) = 611 (28.9%)						
N (fatty liver)	77	180	53	85	161	31
(%)	(72.0)	(58.2)	(76.8)	(80.2)	(70.3)	(41.9)
**Age-sex** adjusted **OR**	**4.96**	**3.14**	**6.70**	**8.48**	**4.78**	**1.60**
(95% CI)	[3.17–7.75]	[2.42–4.08]	[3.74–12.01]	[5.13–14.00]	[3.47–6.59]	[0.98–2.62]
p-value	<.0001	<.0001	<.0001	<.0001	<.0001	0.0605
**Multivariable-** adjusted **OR**	**2.98**	**2.45**	**3.66**	**4.96**	**3.09**	**1.30**
(95% CI)	[1.82–4.87]	[1.83–3.28]	[1.95–6.86]	[2.86–8.60]	[2.17–4.40]	[0.75–2.27]
p-value	<.0001	<.0001	<.0001	<.0001	<.0001	0.3521

Multivariable-adjusted models included age, sex, education, BMI, hypertension, TC/HDL-cholesterol ratio, uric acid, CRP, alcohol intake, smoking status, and physical activity – BMI was not included in **C** because it was used to calculate the index.

Models featuring fatty liver as the main independent variable, defined as two out of three liver enzymes above the upper reference value (disregarding AP as the least influential parameter in our study), yielded highly significant ORs of 2 to 4 in IFG, IGT, NDD and known diabetes, but not in IFG/IGT, which was probably due to very small case numbers in this group. These results remained significant in most subgroups after controlling for confounding variables. Similar numbers were obtained when fatty liver was defined as two out of three enzymes above the upper 75% quartile.

Using the Bedogni Index as an estimation of fatty liver, all persons with pre-diabetes and diabetes were significantly more often affected than the NGT group with a 2 to 8 fold increase of ORs, especially in NDD (OR 8.48, 95% CI: 5.13–14.00) and IFG/IGT (OR 6.70, 95% CI: 3.74–12.01). These numbers even increased slightly after exclusion of subjects with at-risk alcohol intake (data not shown) and remained significant when confounding variables were added to the models.

Interaction terms with fatty liver and sex controlled for age where not significant in models with fatty liver definitions A and B, but in the group with known diabetes using definition C (p = 0.0048). In models including age male participants with fatty liver as defined by the FLI had an OR of 3.21 [95% CI 2.11–4.88] for diabetes, female participants had an OR of 7.92 [95% CI 4.90–12.80]. In fully controlled models the ORs decreased to 2.72 [95% CI 1.71–4.34] and 3.61 [95% CI 2.08–6.27] respectively and the p-value of the interaction term was not significant any more (0.0650).

Similarly to the single enzyme models, all Pseudo-R-Square values were between 0.2 and 0.4 and all Pearson tests and AICs showed appropriate goodness-of-fit.

### Effect modification by BMI

In models stratified for BMI subgroups the association between fatty liver on the one hand and diabetes or pre-diabetes on the other hand was most pronounced in slim men (Def. A, multivariable adjusted OR = 4.94, 95% CI 1.17–20.89, sex*FLI interaction: p = 0.0434, not shown in the table), and pre-obese men and women with significant ORs of around 2 to 7 in age-sex adjusted models and 2 to 5 in multivariable-adjusted models, see table 4. The interaction term sex*FLI was only significant in the group with normal weight using definition A. The effects were smaller, though still significant (except for Def. C mutlivariable adjustment) in the obese group. Interaction by BMI was p<0.0001 in all fatty liver definitions.

**Table 4:** Frequencies and association of fatty liver and glucose metabolism disturbance (pre-diabetes or diabetes) within BMI subgroups.

BMI Group	<25 kg/m^2^Under (n = 10) and Normal weight	25 – <30 kg/m^2^Pre-Obesity	≥30 kg/m^2^Obesity I, II, III
	n = 953	n = 1255	n = 801
**N (%) with Fatty liver**			
**Def. A**	**33 (3.5)**	**76 (6.1)**	**104 (13.0)**
**Def. B**	**122 (12.8)**	**282 (22.5)**	**276 (34.5)**
**Def. C**	**26 (2.7)**	**460 (36.7)**	**712 (88.9)**
**N (%) with**			
**Diabetes**	23 (2.4)	82 (6.5)	124 (15.5)
**Pre-diabetes**	80 (8.4)	237 (18.9)	274 (34.2)
**both**	**103 (10.8)**	**319 (25.4)**	**398 (49.7)**
**Def. A**			
Age-sex- adjusted **OR**	**2.09**	**3.51**	**1.90**
(95% CI)	0.79–5.54	2.07–5.95	1.21–2.98
p-value	0.1387	<0.0001	0.0054
**Def. A**			
Multivariable-adjusted **OR**	**1.92**	**3.14**	**1.62**
(95% CI)	0.71–5.21	1.81–5.42	1.02–2.58
p-value	0.2000	<0.0001	0.0427
**Def. B**			
Age-sex- adjusted **OR**	**2.26**	**2.13**	**1.51**
(95% CI)	1.30–3.93	1.56–2.93	1.10–2.07
p-value	0.0039	<0.0001	0.0108
**Def. B**			
Multivariable-adjusted **OR**	**2.13**	**1.90**	**1.34**
(95% CI)	1.20–3.77	1.37–2.64	0.97–1.87
p-value	0.0099	0.0001	0.0798
**Def. C**			
Age-sex- adjusted **OR**	**7.48**	**2.70**	**1.85**
(95% CI)	2.92–19.18	1.99–3.67	1.10–3.12
p-value	<0.0001	<0.0001	0.0212
**Def. C**			
Multivariable-adjusted **OR**	**5.45**	**2.28**	**1.20**
(95% CI)	2.00–14.85	1.63–3.19	0.68–2.12
p-value	0.0009	<0.0001	0.5239

Multivariable-adjusted models included age, sex, education, hypertension, TC/HDL-cholesterol ratio, uric acid, CRP, alcohol intake, smoking status, and physical activity.

### Stratification by medication use

In order to rule out effect modification from medication use, we conducted a sensitivity analysis calculating models stratified for medication intake and combined probands with diabetes and pre-diabetes to increase statistical power. Participants with pre-diabetes or diabetes were 2.38 times (95% CI 1.90–2.99, p = <.0001) more likely to take any prescription medication. Thus, the subgroup using no medication consisted of 104 (15.6%) probands with diabetes or pre-diabetes and 563 (84.4%) healthy probands. The subgroup using medication included 716 (30.57%) probands with diabetes or pre-diabetes and 1626 (69.43%) healthy probands.

In persons taking no medication, the age and sex adjusted regression effect of fatty liver disease (definition C, Bedogni Index) on glucose disorder had an OR of 5.77 (95% CI 3.40–9.55, p = <0.0001), in persons using medication the respective OR was 4.06 (95% CI 3.30–5.00, p = <0.0001). In multivariable adjusted models, we found ORs 4.29 (95% CI 2.43–7.58, p = <0.0001) and 2.71 (95% CI 2.14–3.44, p = <0.0001) respectively.

P-Interaction by medication use was not significant using any fatty liver definition.

## Discussion

GGT and GPT were significantly elevated in persons with pre-diabetes and diabetes in our study, GOT and AP only in some groups. The effects were sharpened in models using an increase of two out of three enzymes as an estimate of fatty liver and especially in models using the Fatty Liver Index by Bedogni et al.. Up to eight times more KORA participants with fatty liver disease had pre-diabetes or diabetes compared to the healthy reference group. Sensitivity analyses excluding participants with very high liver enzyme values still yielded significant associations of impaired glucose tolerance and liver enzyme concentrations as did models that included a variety of known confounding variables. Likewise, the exclusion of alcohol drinkers did not diminish the effects. We found that particularly in slim men and pre-obese men and women fatty liver disease posed a significant risk for pre-diabetes and diabetes. This supports the idea that fatty liver is a risk factor independent of BMI even though it is more frequent in overweight people. Finally, medication intake diminished the association of NAFLD and glucose metabolism disorder, probably because of a beneficial effect of diabetes medication on the fatty liver, which is able to recover in early stages.

The liver occupies a sentinel position between the alimentary canal and the systemic circulatory system and has wide exposure to toxins and drug metabolites, endotoxins, and infectious agents. Consequently, a wide spectrum of nonhepatic disorders may influence liver enzyme activity. The pattern of liver enzyme abnormalities only provides a first indication of a liver-specific disease [4], [31]. For lack of specific diagnoses of liver disorders in our study we could not exclude patients affected with other liver diseases. Defining elevated liver enzymes as fatty liver disease we found very consistent associations with impaired glucose tolerance and diabetes, even after exclusion of individuals with liver values above certain reference thresholds. These findings match very well with similar analyses in recent literature [8], [9], [12]–[16], [32]–[35] and confirm the fact that the liver is closely involved in glucose metabolism and plays an important role in the development of metabolic disorders.

An earlier evaluation of a different subsample of the MONICA/KORA study [33] already demonstrated a clear correlation of GGT with incident diabetes that was stronger in men than in women. After multivariable adjustment hazard ratios of 1.81 (≥50^th^ vs. <25^th^ percentile) to 4.24 (≥87.5^th^ vs. <25^th^ percentile) across GGT categories were found in men and 1.42 to 2.41 in women respectively over a follow up period of 14.7 years. Our new analysis also took into account further relevant liver enzymes and pre-diabetic states.

The prospective Japanese Hisayama Study found that the age-adjusted cumulative incidence of diabetes increased significantly from lower to higher quartiles of GGT and GPT. The association remained significant after controlling for comprehensive risk factors in both sexes. GOT results were not that clear [32]. Similarly, data from the British Women's Health and Heart Study revealed associations between GGT and GPT with fasting glucose, fasting insulin and HbA1c as measures of glucose homeostasis in 3086 without and 308 women with diabetes. Interestingly, these associations did not differ substantially between obese and non-obese non-diabetic women. The authors cite that liver fat content is not stringently correlated with intra-abdominal or subcutaneous fat depots, thus explaining that non-obese individuals may occasionally be more affected by fatty liver and interrelated metabolic impairments than obese individuals [12]. An analysis using data from the Framingham Heart Study also confirmed that fatty liver is associated with dysglycemia independent of visceral adipose tissue and other obesity measures [35].

In a meta-analysis with participants of the British Women's Health and Heart Study, the research group confirmed their first findings, and added ultrasound examinations of the liver. Ultrasonography-diagnosed NAFLD was associated with a doubling in the risk of incident diabetes and both GGT and GPT predicted diabetes. [8]

Hanley et al. used data from the Insulin Resistance Atherosclerosis Study conducted in the US and described positive associations of GOT and GPT with incident type 2 diabetes, fasting insulin, waist circumference and fasting glucose, concluding that GOT and GPT independently predict type 2 diabetes [9].

All four potentially relevant liver enzymes were studied in the population-based Mexico City Diabetes Study involving 1,441 men and women in whom serum enzyme levels were ≤3 SDs of the mean population value. At the 7 years follow-up 94 subjects had developed diabetes and 93 impaired glucose tolerance. Upon including known confounding factors, GPT and GGT values were significantly associated with both IGT and diabetes, AP with diabetes only and GOT with IGT only. Raised GGT alone was associated with all the features of the metabolic syndrome. The authors concluded that raised GGT is an independent predictor of deterioration of glucose tolerance to IGT or diabetes and may reflect hepatic steatosis and oxidative stress [13].

Mohan et al. investigated the relationship in urban south Indian subjects, applying OGT test and ultrasonography of the liver and concluded that NAFLD was present in a third of the included subjects and increased significantly with increasing severity of glucose tolerance (NGT: 22.5%, IFG: 27.3%, IGT: 32.4%, IGT/IFG: 33.0%, type 2 diabetes: 54.5%) [34]. These numbers go rather well together with our quartile definition, but we did not find such a perfect continuous rise in frequency (NGT: 18.7%, IFG: 34.6%, IGT: 33.0%, IGT/IFG: 33.3%, NDD: 42.5%, known diabetes: 25.8%).

The theory has been raised that analogous to Palmipedes (migratory fowl) fatty liver could be a natural adaptation to facilitate survival in a cold and resource-scarce environment. This would explain why fatty liver is not a uniform feature of the metabolic syndrome but varies remarkably within ethnicities and individuals [36] and why obese individuals displaying the metabolically healthy but obese (MHO) phenotype present favourable levels of liver enzymes [37].

To put it into a nutshell, the results of the population-based KORA study are not completely novel, but nevertheless make a valuable contribution to the present body of acquired knowledge. The Fatty Liver Index has not been used exhaustively in the past. However, we found that the FLI is a useful approximation in a population-based study that cannot collect biopsy data due to ethical concerns. The Fatty Liver Index has also been used in the French D.E.S.I.R Study in association with incident diabetes [18]. The authors found that a FLI ≥70 in comparison with a FLI <20 had an age adjusted OR of 9.33 (95% CI 5.05–17.25) for men and 36.72 (95% CI 17.12–78.76) for women. They concluded that the FLI is predictive of diabetes in men and women independently of traditional risk factors and suggested that the index should be used by hepatologists to better identify patients at high risk of diabetes.

Similarly, in the Italian RISC study a significant reduction of insulin sensitivity was found in middle-aged non-diabetic subjects with a FLI Score greater than or equal 60 [19].

Our study shows, that even though liver values are differently distributed in men and women, the risk of elevated enzymes on metabolic disorders is equally present in both sexes. BMI is strongly associated with NAFLD, however, the negative influence of NAFLD on glucose metabolism (or vice versa) is definitely detectable in slender and pre-obese individuals.

### Study limitations

In the population based KORA study only liver enzyme measurements as surrogates of fatty liver disease were accessible, ultrasounds could unfortunately not be implemented and biopsies can not be done in epidemiological studies in mostly healthy subjects. It has been stated that by using liver enzymes alone the true prevalence of NAFLD may be underestimated by more than 50%. Belentani & Marino report that up to 79% of patients diagnosed with NAFLD actually present normal aminotransferase levels [2]. 20% of young patients with non-alcoholic steatohepatitis (NASH) and/or fibrosis diagnosed by biopsies and histological examinations also have normal GPT values at the time of biopsy [38]. By using the Bedogni-Index in addition to the liver enzymes, we tried to improve our assessment of NAFLD cases and found that the association between the index and pre-diabetes or diabetes respectively was remarkably stronger than for the liver enzymes alone.

We did not omit participants with significant consumption of alcohol from our main models and therefore cannot exclude, that some cases of fatty liver were due to alcohol abuse, but sensitivity analyses without the participants in question showed consistent results. Unfortunately, verified information on viral hepatitis status that could have affected liver enzymes was not available in the present study.

Moreover, we only looked at cross sectional data of the KORA study and are thus not able to give evidence for temporal causality of the associations described above.

In conclusion elevated GGT and GPT – values as well as estimates of fatty liver disease are significantly associated with pre-diabetes and diabetes and thus easily available and relatively inexpensive first indicators of a disturbed glucose metabolism.
